# Miscellany

**Published:** 1909-07

**Authors:** 


### MISCELLANY.

THE United States Civil Service Commission announces an examination on July 14, 1909, at the usual places to secure eligibles from which to make certification to fill a vacancy in the position of pathologist (male), $2,000 per annum, Freedmen's Hospital, Washington, D. C., and vacancies requiring similar qualifications as they may occur in that hospital.

The Commission also announces an examination on July 21, 1909, at the usual places to secure eligibles from which to make certification to fill vacancies as they occur in the position of physician, at $150 per month, in the Panama Canal Service. Applicants should at once apply either to the United States Civil Service Commission, Washington, D. C., or to the secretary of the local board of examiners for application Form 1312. The medical certificate in Form 1312 must be filled in by a reputable practicing physician other than the applicant. No application will be accepted unless properly executed and filed with the Commission at Washington prior to the hour of closing business on July 10, 1909.

